# Glucose Fluctuations Aggravate Myocardial Fibrosis *via* the Nuclear Factor-κB-Mediated Nucleotide-Binding Oligomerization Domain-Like Receptor Protein 3 Inflammasome Activation

**DOI:** 10.3389/fcvm.2022.748183

**Published:** 2022-05-03

**Authors:** Zhen-Ye Zhang, Shi-Peng Dang, Shan-Shan Li, Ying Liu, Miao-Miao Qi, Ning Wang, Ling-Feng Miao, Ying Wu, Xiao-Yan Li, Chun-Xin Wang, Ling-Ling Qian, Ru-Xing Wang

**Affiliations:** ^1^Department of Cardiology, The Affiliated Wuxi People’s Hospital of Nanjing Medical University, Wuxi, China; ^2^Department of Medical Laboratory, The Affiliated Wuxi People’s Hospital of Nanjing Medical University, Wuxi, China

**Keywords:** diabetes, glucose fluctuation, myocardial fibrosis, nuclear factor-κB, inflammasome

## Abstract

**Background:**

Glucose fluctuations may be associated with myocardial fibrosis. This study aimed to investigate the underlying mechanisms of glucose fluctuation-related myocardial fibrosis.

**Methods:**

Streptozotocin (STZ)-injected type 1 diabetic rats were randomized to five groups: the controlled blood glucose (CBG) group, uncontrolled blood glucose (UBG) group, fluctuated blood glucose (FBG) group, FBG rats injected with 0.9% sodium chloride (NaCl) (FBG + NaCl) group, and FBG rats injected with MCC950 (FBG + MCC950) group. Eight weeks later, left ventricular function was evaluated by echocardiography and myocardial fibrosis was observed by Masson trichrome staining. The primary neonatal rat cardiac fibroblasts were cultured with different concentrations of glucose *in vitro*.

**Results:**

The left ventricular function was impaired and myocardial fibrosis was aggravated most significantly in the FBG group compared with the CBG and UBG groups. The levels of interleukin (IL)-1β, IL-18, transforming growth factor-β1 (TGF-β1), collagen type 1 (collagen I), nuclear factor (NF)-κB, and nucleotide-binding oligomerization domain-like receptor protein 3 (NLRP3) inflammasome were significantly increased in the FBG group. *In vitro*, the inhibition of NF-κB and inflammasome reversed these effects. *In vivo*, NLRP3 inhibition with MCC950 reversed left ventricular systolic dysfunction and myocardial fibrosis induced by glucose fluctuations.

**Conclusion:**

Glucose fluctuations promote diabetic myocardial fibrosis by the NF-κB-mediated inflammasome activation.

## Introduction

Diabetes mellitus is regarded as an important contributor to the development of cardiovascular disease (1, 2). Several studies have demonstrated that diabetic cardiovascular complications are not only related to persistent high blood glucose but also blood glucose fluctuations (3, 4). Glucose fluctuations have been reported to aggravate cardiac fibrosis (5). However, the potential mechanisms by which glucose fluctuations promote myocardial fibrosis have to date remained largely unexplored.

Accumulated evidence has revealed the important role of inflammasome in diabetic myocardial fibrosis (6). Inflammasome, a multiple-protein complex, is composed of a nucleotide-binding oligomerization domain-like receptor (NLR) protein, an apoptosis-associated speck-like protein containing a caspase-recruitment domain (ASC) and procaspase-1 (7). NLRP3, one of the NLRs, functions as a sensor and a crucial regulator in metabolic disorders (8). In active state, NLRP3 interacts with ASC and subsequently facilitates the activation of caspase-1, leading to processing several pro-inflammatory cytokines into their mature forms, such as interleukin (IL)-1β and IL-18 (9). Increased inflammatory cytokines can aggravate the cardiomyocyte dysfunction and promote myocardial fibrosis (10, 11). However, whether glucose fluctuations lead to the activation of NLRP3 inflammasome remains unclear.

Nuclear factor (NF)-κB, a ubiquitous member of the family of transcription factors, is involved in regulating the transcription of the NLRP3 gene, leading to increasing NLRP3 expression and activating inflammasome (12). In normal cells, NF-κB p65 subunit combines with its inhibitory subunit (IκB) to keep in an inactive state. IκB dissociates from p65 after phosphorylation by IκB kinase (IKK), which leads to NF-κB entering the nuclear and mediating transcription (13, 14). In response to high glucose, NF-κB can be activated and participate in various kinds of pathologic and physiological processes (15). Moreover, it has been reported that NF-κB was activated not only in diabetic rats, but also in rats with fluctuated blood glucose *in vivo* (5, 16). In the present study, we demonstrated that the glucose fluctuation-induced NF-κB-mediated activation of NLRP3 inflammasome contributes to the promotion of diabetic myocardial fibrosis.

## Materials and Methods

### Establishment of Experimental Animal Models

Male Sprague–Dawley (SD) rats (6–8 weeks) weighting 150–200 g (*n* = 36) were purchased from Changzhou Cavens Laboratory Animal Company in China. The rats were raised at a 12 h light/dark cycle under standard of care specific pathogen-free (SPF) conditions (temperature 23 ± 1°C and humidity 55–65%). The type 1 diabetic rat model was established as previously reported (17). Rats were received an intraperitoneal injection of streptozotocin (STZ; Sigma-Aldrich; 60 mg/kg) and after 1 week, rats with a blood glucose concentration > 16.7 mmol/L were enrolled in this study. Rats were randomly divided into five groups: the controlled blood glucose (CBG) group, uncontrolled blood glucose (UBG) group, fluctuated blood glucose (FBG) group, FBG rats injected with 0.9% sodium chloride (NaCl) (FBG + NaCl) group, and FBG rats injected with MCC950 (FBG + MCC950) group. For the CBG group, the diabetic rats received injections of long-acting insulin (insulin glargine; Sanofi-Aventis; 20 IU/kg) twice a day. For the UBG group, the diabetic rats were delivered sufficient food and water. For the FBG group, following 24h of starvation, the diabetic rats were injected regular insulin (insulin human; Novo Alle; 0.5 IU/kg) if blood glucose level was > 5.5 mmol/L. Thereafter, rats were fed adequate food to increase their blood glucose. The FBG + MCC950 group rats were treated with starvation and consumption as the same as the FBG group rats, and were intraperitoneally injected with MCC950 (3 mg/kg, Selleck, S7809) daily. While, FBG + NaCl group rats were intraperitoneally injected with 0.9% NaCl as a placebo. Eight weeks later, rats were sacrificed for experiments after being injected intraperitoneally with sodium pentobarbital (60 mg/kg) and the hearts were harvested and stored at −80°C. The ratios of heart weight/body weight (HW/BW) were calculated. All animal protocols were approved by the Institutional Animal Care and Use Committee (IACUC) of Nanjing Medical University (IACUC-1712028).

### Cardiac Function Measurement

Cardiac function was evaluated by echocardiography (Philip, ie33). Rats were anesthetized with isoflurane (2%). Left ventricular percent ejection fraction (EF), left ventricular fractional shortening (FS), left ventricular internal diameter at end-diastole (LVIDd), and left ventricular internal diameter at end-systole (LVIDs) were measured to evaluate left ventricular systolic function. All measurements were made by an observer who was blinded to the identity of the tracings. Data of EF, FS, LVIDd, and LVIDs were averaged over six consecutive cardiac cycles.

### Histological Section

Hearts were harvested and fixed in 4% paraformaldehyde. After embeddeding in paraffin, hearts were cut into 5 μm slices and stained with Masson trichrome staining (Nanjing Jiancheng Bioengineering Institute, D026). For hematoxylin and eosin (HE) staining, the slices were stained with hematoxylin (Servicebio, G1004) and eosin (Servicebio, G1001). For immunohistochemistry, the slices were incubated with anti-α-SMA antibody (Abcam, a5694) over night at 4°C. Next, the slices were incubated with Diaminobenzidine solution (Zsbio, ZLI-9018) for 10 min followed by counterstaining with hematoxylin (Beyotime, C0107). Following dehydration, coverslips were mounted with neutral balsam (HUSHI, 10004160). Finally, the images were captured under a light microscope (Leica Microsystems). The α-SMA protein positive signals were brown in color.

### Glucose Monitoring and ELISA of IL-1β

The blood glucose levels of all diabetic rats were measured with a blood glucose meter (Roche, Switzerland) daily. Eight weeks later, rats were sacrificed and blood samples were collected from the inferior vena cava. The IL-1β levels were detected using IL-1β ELISA kit (Invitrogen, BM3630).

### Primary Neonatal Rat Cardiac Fibroblasts Culture

In order to isolate primary neonatal rat cardiac fibroblasts (NRCFs), neonatal SD rats (1–3 days old) were purchased from Changzhou Cavens Laboratory Animal Company in China. The hearts were quickly separated and digested with 0.125% trypsin (Gibco, 25200072) and 0.1% collagenase (Worthington-Biochem, LS004176) by a series of 5 incubations at 37°C. The digested tissue was then prepared into cell suspension, and plated in DMEM for primary cell isolation (Thermo, 88287) with 10% FBS (Gibco, 12664025). After an hour, the unattached cells were removed and new medium was added. Cells were seeded at 1 × 10^6^/100-mm culture dishes (Corning, 430167) and initially incubated at 37°C and 5% CO_2_.

### Glucose Fluctuations Model *in vitro*

The glucose fluctuation model *in vitro* was established as reported previously. In short, primary NRCFs were divided into three groups: the normal glucose (NG) group, the high glucose (HG) group and the glucose fluctuations (GF) group. Cells in the NG group were incubated in DMEM containing 5.5 mmol/L glucose. Cells in the HG group were incubated in DMEM containing 25mmol/L glucose. Cells in the GF group were incubated for 72 h in DMEM alternating between 5.5 and 25 mmol/L glucose every 12 h. When cells were treated with the IKK inhibitor TPCA-1 (Sigma, T1452-1MG) (0.5 μM), these agents were added at the beginning and remained present throughout the experiment.

### SiRNA Transfection

Primary neonatal rat cardiac fibroblasts were cultured with serum-free medium and subsequently transfected with NLRP3 siRNA (GenePharma). The sequence of siRNA duplexes targeting NLRP3 was as follows: sense 5′-CCAGGAGAGAACUUCUUAUTT-3′, anti-sense 5′-AUA AGAAGUUCUCUCCUGGTT-3′. After 6 h incubation, the media containing siRNA was replaced by DMEM containing 10 % FBS.

### Quantitative Real-Time PCR

Total RNA was extracted from hearts and was subsequently reverse-transcribed using a Reverse Transcription System (Takara, DRR036A). Quantitative PCR was performed using SYBR Green PCR mix (Roche, 4913914001) on an ABI Prism 7500HT Sequence Detection System (Applied Biosystems). The β-actin was used as an internal control to normalize for differences in the amount of total RNA in each sample. Primer pairs for cDNA amplification were as follows: AAGAAGGACCAGCCAGAGTG (forward) and TGGGTGTAGCGTCTGTTGAG (reverse) for NLRP3; CAACAATTCCTGGCGTTACCT (forward) and AGCCCTGTATTCCGTCTCCTT (reverse) for TGF-β1; CTACAGCACGCTTGTGGATG (forward) and GATTGGGA TGGAGGGAGTTTAC (reverse) for collagen I; TCCAGT CAGGCTTCCTTGTG (forward) and CGAGATGCTGCTG TGAGATT (reverse) for IL-1β; ACCGCAGTAATACGGAGCAT (forward) and CTGGGATTCGTTGGCTGTTC (reverse) for IL18; AGATTACTGCCCTGGCTCCTA (forward) and CCTGCTTGCTGATCCACATCT (reverse) for β-actin.

### Western Blot

Heart tissues were homogenized and lysed with ice-cold RIPA buffer (Pierce, 89900) containing protease and phosphatase inhibitors (Roche, 04693159001). The lysates were fractionated by SDS-PAGE and transferred onto polyvinylidene difluoride (PVDF) membranes (Amersham Biosciences). The PVDF membranes were incubated with the specific primary antibodies to α-tubulin (Cell Signaling Technology, 2144), GAPDH (Cell Signaling Technology, 2118), NF-κB p65 (Cell Signaling Technology, 8242), p-NF-κB p65 (Cell Signaling Technology, 3033), NLRP3 (LifeSpan BioSciences, LS-C334192), ASC (Novus Biologicals, NBP1-78977), cleaved caspase-1 (Novus Biologicals, NB100-56565), TGF-β1 (Abcam, ab179695), collagen I (Abcam, ab34710) and collagen III (Abcam, ab7778). Immunoblot bands were quantified by densitometry using ImageJ. Densities were normalized to control treatment and relative folds were normalized to α-tubulin or GAPDH.

### Confocal Microscopy

Neonatal rat cardiac fibroblasts were fixed with 4% paraformaldehyde, permeabilized with 0.1% (vol/vol) Triton X-100 (Sigma-Aldrich, T9284), blocked with 2% BSA (Santa Cruz, sc-2323). For the assay of NF-κB nucleation, cells were incubated with anti-NF-κB p65 (Cell Signaling Technology, 8242). Cells were washed with PBST and incubated with an anti-rabbit IgG (H + L) F (ab’)_2_ fragment secondary antibody (Cell Signaling Technology, 4412) and DAPI. For the assay of intracellular α-SMA and TGF-β1, cells were incubated with anti-α-SMA (Abcam, a5694) and TGF-β1 (Abcam, ab179695) respectively, and then incubated with an anti-rabbit IgG (H + L) F (ab’)_2_ fragment secondary antibody. For the measurement of intracellular ASC and NLRP3, cells were incubated with the mixture of anti-NLRP3 antibody (LifeSpan BioSciences, LS-C334192, raised in rabbit) and anti-ASC antibody (Santa, sc-514414, raised in mouse) overnight. After being washed with PBST, cells were incubated with the mixture of anti-rabbit Cy3-labeled secondary antibody (Jackson, 111-165-003) and anti-mouse FITC-labeled secondary antibody (Jackson, 115–095-003) together with DAPI. For immunofluorescence staining of heart tissues, the rat hearts were harvested and fixed with 4% paraformaldehyde. Following OCT paraffin, hearts were cut into 4 μm sections. The heart sections were analyzed to detect for NLRP3 and ASC with the above mentioned antibodies. The identical procedure of permeation, blocking and incubating the antibodies was performed on myocardial tissues.

### Statistical Analysis

Data were presented as mean ± SEM. One-way ANOVA with *post hoc* LSD analysis was used to compare data from multiple groups. Student’s *t*-test was used to compare data between two groups. Statistical significance was defined as *P* < 0.05. Statistical analysis was performed with GraphPad Prism 5 and SPSS 22.0.

## Results

### Glucose Fluctuations Aggravated the Impairment of Left Ventricular Systolic Function

**FIGURE 1:**
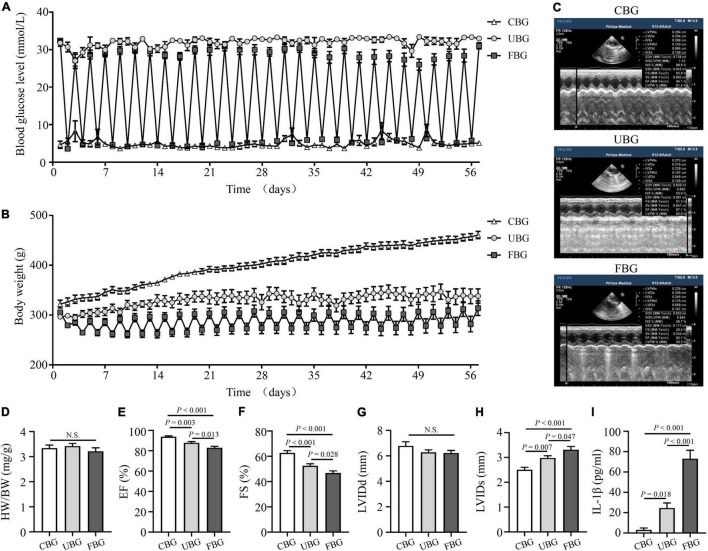
Glucose fluctuations promoted rat left ventricular systolic dysfunction. **(A)** Levels of blood glucose in the CBG, UBG, and FBG groups (*n* = 8 per group). **(B)** Body weight in the three groups (*n* = 8 per group). **(C,D)** Representative echocardiographic images of rats and heart weight/body weight (HW/BW) in the three groups (*n* = 8 per group). **(E–H)** Representative echocardiographic images of rats, left ventricular ejection fraction (% EF), left ventricular fractional shortening (% FS), left ventricular internal diameter at end-diastole (LVIDd), and left ventricular internal diameter at end-systole (LVIDs) examined by echocardiography (*n* = 8 per group). **(I)** IL-1β level in the three groups (*n* = 6 per group). N.S.: No significance.

Blood glucose levels of rats in the CBG group were controlled by long-acting insulin to maintain approximately 5 mmol/L (Figure 1A). Blood glucose levels of rats in the FBG group with repetitive starvation and additional insulin injections fluctuated between 5 and 25 mmol/L, while blood glucose levels of rats in the UBG group were always > 25 mmol/L. Body weight gains of rats in the UBG and FBG groups were significantly less than those in the CBG group. The fluctuations of body weights in the FBG group were associated with repetitive starvation (Figure 1B). After 8 weeks, the echocardiography representative images were shown in Figure 1C and the HW/BW ratios showed no differences among the three groups (Figure 1D). The LVEF (%) was decreased in the UBG group (87.70 ± 1.27) when compared with the CBG group (93.70 ± 0.86), which was more pronounced in the FBG group (82.88 ± 1.54) (Figure 1E). We noted a downward trend of FS (%) in the UBG group (52.44 ± 1.63) compared with the CBG group (62.64 ± 1.82), which was to a greater extent in the FBG group (46.68 ± 1.73) (Figure 1F), which suggested that glucose fluctuations promoted the impairment of left ventricular systolic function in the diabetic rats. There were no differences of LVIDd among the three groups (Figure 1G). However, LVIDs was increased in the UBG group compared with the CBG group, which was more pronounced in the FBG group (Figure 1H). In addition, the IL-1β level was significantly increased in the FBG group (Figure 1I).

### Glucose Fluctuations Aggravated Myocardial Fibrosis

**FIGURE 2:**
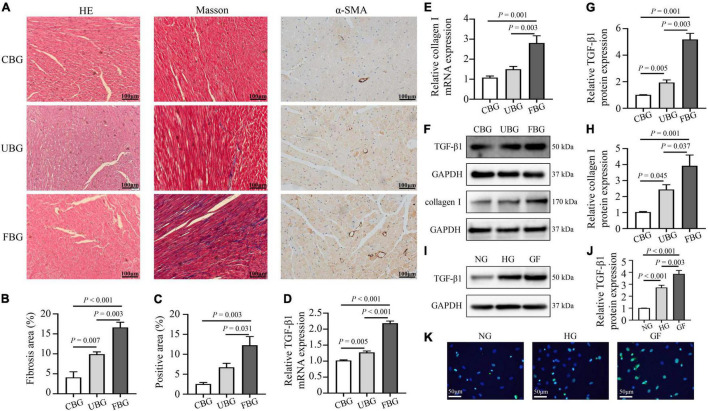
Glucose fluctuations promoted myocardial fibrosis. **(A–C)** Representative images of hematoxylin and eosin (HE) staining for myocardial tissue in the CBG, UBG, and FBG groups (*n* = 3 per group). Representative images of myocardial fibrosis and fibrosis area (%) in the CBG, UBG, and FBG groups (*n* = 3 per group). Representative images of immunohistochemistry staining of α-SMA and positive area (%) in the three groups (*n* = 3 per group). **(D,E)** The mRNA levels of TGF-β1 and collagen I in rat hearts of the three groups (*n* = 4 per group). **(F–H)** The protein expressions of TGF-β1 and collagen I in rat hearts of the three groups (*n* = 4 per group). **(I,J)** The protein expressions of TGF-β1 in NRCFs of the NG, HG and GF groups (*n* = 4 per group). **(K)** Immunofluorescence staining of intracellular TGF-β1 in the NG, HG, and GF groups (*n* = 3 per group).

Figures 2A–C showed the HE staining, Masson trichrome staining and immunohistochemistry staining of α-SMA in heart tissues. HE staining showed that most of the cardiomyocytes in the CBG group were arranged in order, with clearly layered structure, clear and blue stained nucleus and evenly stained cytoplasm. However, myocardium in the UBG and FBG group were arranged in disorder, with vacuolar denature, deformity in size and shape of cytoplasm and elevated cross-section surface areas. To investigate the effects of glucose fluctuations on myocardial fibrosis, rat heart sections were stained with Masson trichrome staining and myocardial fibrosis was significantly increased in diabetic rats of the FBG group. Moreover, α-SMA expression in rat hearts evaluated by immunohistochemistry staining was significantly increased in FBG group. Both the mRNA and protein levels of TGF-β1 and collagen I were significantly upregulated in the FBG group (Figures 2D–H), and these results indicated that glucose fluctuations promote myocardial fibrosis in the diabetic rats. Additionally, the expression of TGF-β1 was significantly upregulated in NRCFs with fluctuated glucoses (Figures 2I–K).

### Glucose Fluctuations Promoted the Activation of Nucleotide-Binding Oligomerization Domain-Like Receptor Protein 3 Inflammasome

**FIGURE 3:**
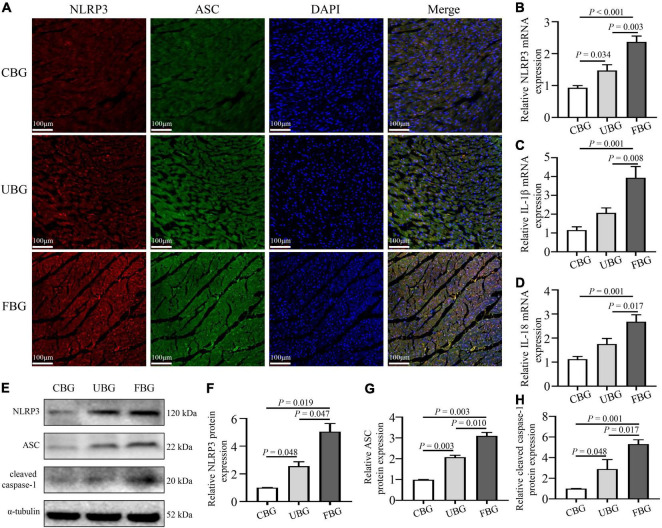
Glucose fluctuations promoted the activation of NLRP3 inflammasome. **(A)** Immunofluorescence staining of NLRP3-ASC interaction in rat hearts. The heart sections were labeled with anti-NLRP3 (red), anti-ASC (green), and DAPI (blue) (*n* = 3 per group). **(B–D)** The mRNA levels of NLRP3, IL-1β and IL-18 in rat hearts of the three groups (*n* = 4 per group). **(E–H)** The protein expressions of NLRP3, ASC and cleaved caspase-1 in rat hearts of the three groups (*n* = 4 per group).

To confirm the activation of NLRP3 inflammasome induced by glucose fluctuations, immunofluorescence staining was used to evaluate NLRP3-ASC interactions in heart tissues. The results showed that NLRP3-ASC interactions were significantly enhanced in the FBG group (Figure 3A). To determine the role of inflammasome in glucose fluctuation-induced myocardial fibrosis, the expressions of important proteins that formed inflammasome, such as NLRP3, ASC, and cleaved caspase-1 were tested. The mRNA levels of NLRP3, IL-1β, and IL-18 were increased significantly in the FBG group (Figures 3B–D). Compared with the CBG group, the expressions of these proteins were higher in UBG group, which were more pronounced in the FBG group (Figures 3E–H). Collectively, these results indicated that glucose fluctuations promoted the activation of NLRP3 inflammasome in the diabetic rats.

### Exposure to Glucose Fluctuations Promoted Myocardial Fibrosis via the Activation of Nucleotide-Binding Oligomerization Domain-Like Receptor Protein 3 Inflammasome

**FIGURE 4:**
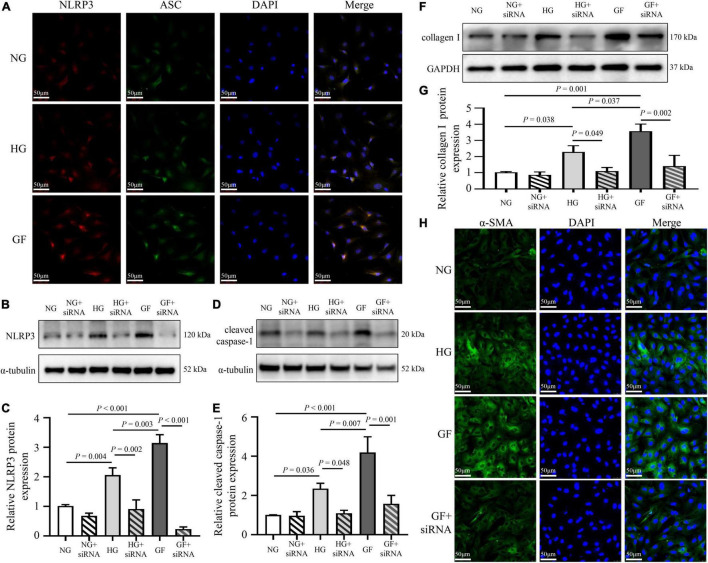
Role of inflammasome in glucose fluctuation-induced myocardial fibrosis. **(A)** Immunofluorescence staining of intracellular NLRP3–ASC interaction examined by confocal microscopy. The NRCFs were labeled with anti-NLRP3 (red), anti-ASC (green), and DAPI (blue) (*n* = 3 per group). **(B–G)** After siRNA targeting NLRP3 transfection, the protein expressions of NLRP3, cleaved caspase-1 and collagen I were measured by western blot (*n* = 3 per group). **(H)** Representative images of immunofluorescence staining of intracellular α-SMA in the NG, HG, GF, and GF + siRNA groups (*n* = 3 per group).

To analyze the effect of glucose fluctuations on myocardial fibrosis *in vitro*, NRCFs were exposed to different glucose concentrations. To characterize the formation of the inflammasome in NRCFs, immunofluorescence assay was used to evaluate intracellular NLRP3–ASC interactions. The NLRP3–ASC interaction was obviously observed in the cells with fluctuated glucose concentrations (Figure 4A). To further determine the role of NLRP3 inflammasome on myocardial fibrosis, NLRP3 siRNA was transfected into NRCFs. After transfection, protein level of NLRP3 was decreased, high glucose and glucose fluctuations induced upregulation of cleaved caspase-1was inhibited (Figures 4B–E). Moreover, the inhibition of NLRP3 reversed the upregulation of collagen I and α-SMA (Figures 4F–H). These results suggested that inflammasome was activated in response to glucose fluctuations and played an important role in the regulation of myocardial fibrosis.

### Glucose Fluctuations Promoted Nucleotide-Binding Oligomerization Domain-Like Receptor Protein 3 Inflammasome-Induced Myocardial Fibrosis via Activating NF-κB

**FIGURE 5:**
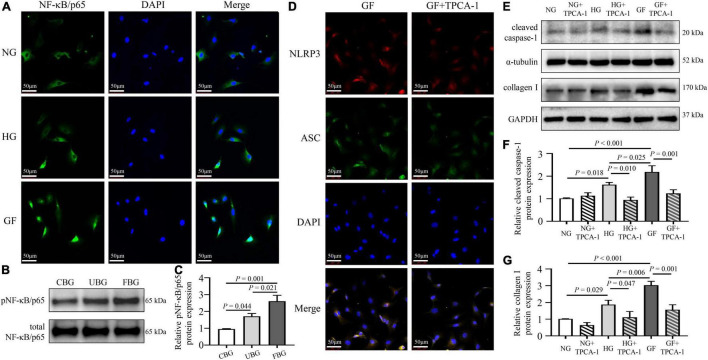
Role of NF-κB in glucose fluctuation-induced inflammasome-related myocardial fibrosis. **(A)** Immunofluorescence staining of NF-κB/p65 nucleation (*n* = 3 per group). **(B,C)** The phosphorylation level of NF-κB/p65 in rat hearts of the three groups (*n* = 4 per group). **(D)** Immunofluorescence staining of intracellular NLRP3–ASC interaction in the GF and GF + TPCA-1 groups. The NRCFs were labeled with anti-NLRP3 (red), anti-ASC (green), and DAPI (blue) (*n* = 3 per group). **(E–G)** After application of TPCA-1 (0.5 μM), the protein expressions of cleaved caspase-1 and collagen I were measured by western blot (*n* = 3 per group).

To investigate the mechanism of inflammasome activation by glucose fluctuations, immunofluorescence assay was used to evaluate the nuclear translocation of NF-κB. The nucleation of NF-κB/p65 was obviously observed in NRCFs with glucose fluctuations compared with normal and high glucose (Figure 5A). The phosphorylation level of NF-κB/p65 was significantly increased in the GF group (Figures 5B,C). After application of IKK inhibitor TPCA-1 (0.5 μM), the NLRP3-ASC interaction was weakened in NRCFs of the GF group (Figure 5D). Moreover, after applying TPCA-1, the expressions of cleaved caspase-1 and collagen I declined, respectively, in NRCFs of the HG and GF groups (Figures 5E–G). These data indicated that the nucleation of NF-κB was responsible for the activation of inflammasome after exposure to fluctuated glucose concentrations.

### Nucleotide-Binding Oligomerization Domain-Like Receptor Protein 3 Inhibition With MCC950 Reversed Left Ventricular Systolic Dysfunction and Myocardial Fibrosis Induced by Glucose Fluctuations

**FIGURE 6:**
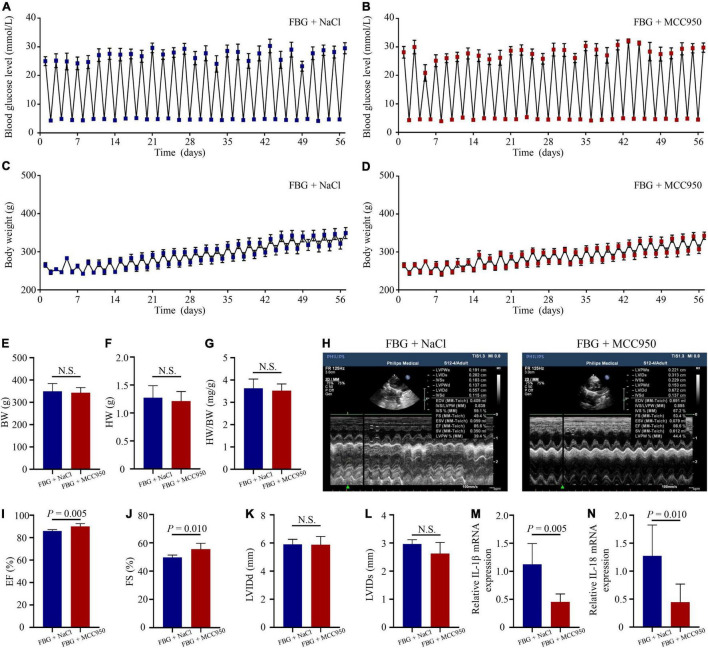
NLRP3 inhibition with MCC950 reversed rat left ventricular systolic dysfunction induced by glucose fluctuations. **(A,B)** Levels of blood glucose in the FBG + NaCl and FBG + MCC950 groups (*n* = 6 per group). **(C,D)** Body weights in the FBG + NaCl and FBG + MCC950 groups (*n* = 6 per group). **(E–G)** Body weights, heart weights and heart weight/body weight ratios of rats in the FBG + NaCl and FBG + MCC950 groups (*n* = 6 per group). **(H–L)** Representative echocardiographic images of rats, LVEF (%), LVFS (%), LVIDd and LVIDs in the FBG + NaCl and FBG + MCC950 groups (*n* = 6 per group). **(M,N)** Relative mRNA levels of IL-1β and IL-18 in the FBG + NaCl and FBG + MCC950 groups (*n* = 6 per group). N.S.: No significance.

**FIGURE 7:**
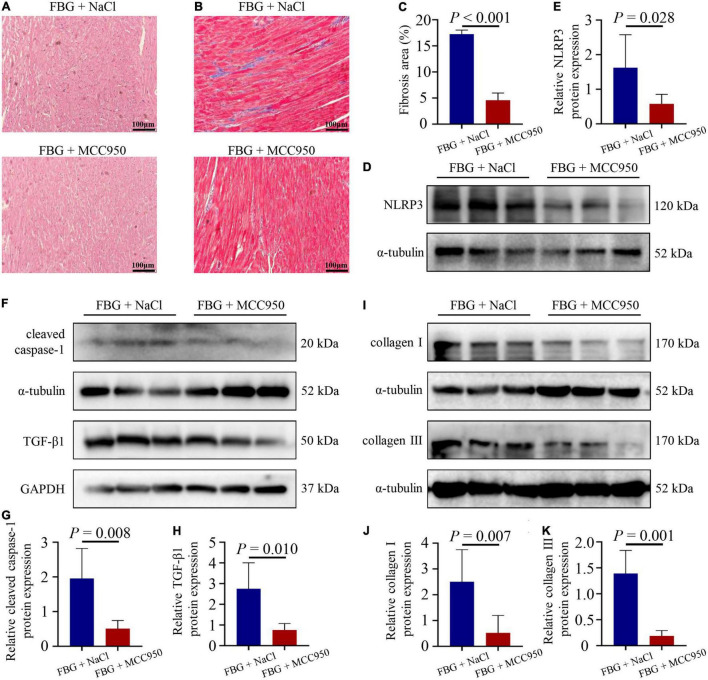
NLRP3 inhibition with MCC950 reversed myocardial fibrosis induced by glucose fluctuations. **(A)** Representative images of hematoxylin and eosin (HE) staining for myocardial tissue in the FBG + NaCl and FBG + MCC950 groups (*n* = 3 per group). **(B–C)** Representative images of myocardial fibrosis and fibrosis area (%) in the FBG + NaCl and FBG + MCC950 groups (*n* = 4 per group). **(D–K)** The protein expressions of NLRP3, cleaved caspase-1, TGF-β1, collagen I and collagen III in the FBG + NaCl and FBG + MCC950 groups (*n* = 6 per group).

The blood glucose levels and the body weights of rats in the FBG + NaCl and FBG + MCC950 groups are shown in Figures 6A–D. There were no differences of body weights, heart weights, and HW/BW ratios among the two groups (Figures 6E–G). The LVEF (%) was increased in the FBG + MCC950 (89.92 ± 1.02) when compared with the FBG + NaCl group (85.83 ± 0.54) (Figures 6H,I). Moreover, the FS (%) was also increased in the FBG + MCC950 (55.52 ± 1.69) when compared with the FBG + NaCl group (49.72 ± 0.68) (Figure 6J). The LVIDd and LVIDs showed no differences among the two groups (Figures 6K,L). Compared with the FBG + NaCl group, both the mRNA levels of IL-1β and IL-18 were decreased in the FBG + MCC950 group (Figures 6M,N). The representative images of HE staining in the FBG + NaCl and FBG + MCC950 groups were shown in Figure 7A. As shown in Figures 7B,C, myocardial fibrosis was significantly declined in diabetic rats of the FBG + MCC950 group. Moreover, the protein expressions of NLRP3, cleaved caspase-1, TGF-β1, collagen I and collagen III were decreased in the FBG + MCC950 group (Figures 7D–K).

## Discussion

Several studies have reported that glucose fluctuations might be associated with myocardial fibrosis and cardiac dysfunction (3, 5); however, the precise mechanisms are still unknown. The present study focused on investigating the mechanism related to myocardial fibrosis caused by glucose fluctuations. The main findings of this study were: (1) Blood glucose fluctuations promoted myocardial fibrosis by activating inflammasome. (2) Glucose fluctuations aggravated inflammasome-related myocardial fibrosis by activating NF-κB.

Compared with the CBG group, the EF (%) and FS (%) data measured by echocardiography were decreased in the UBG group, and to a greater extent in the FBG group. Furthermore, Masson trichrome staining data showed that myocardial fibrosis was obviously observed in diabetic rats with glucose fluctuations, along with the upregulation of TGF-β1, α-SMA and collagen I expressions. These results suggested that glucose fluctuations accelerated cardiac systolic dysfunction and myocardial fibrosis more significantly than persistent hyperglycemia.

Inflammasome, a group of protein complexes, is involved in myocardial fibrosis in diabetes (8, 11). NLRP3 interacts with procaspase-1 via the adaptor protein ASC. Furthermore, the formation of the NLRP3 inflammasome complex contributes to caspase-1 activation and release of pro-inflammatory cytokines including IL-1β and IL-18, which results in pro-inflammatory cytokines maturation (18). In this study, we found that the expression of NLRP3, the levels of IL-1β and IL-18 were significantly increased after exposure to glucose fluctuations. Moreover, increased NLRP3-ASC interaction induced by fluctuated glucose was obviously observed by immunofluorescence staining both *in vivo* and *in vitro*. To further confirm the role of NLRP3 inflammasome in glucose fluctuation-mediated myocardial fibrosis, we used siRNA to inhibit the expression of NLRP3 in NRCFs. Moreover, we applied the inhibitor of NLRP3 inflammasome *in vivo*. Left ventricular systolic dysfunction and myocardial fibrosis were reversed in the FBG + MCC950 group rats, compared with those in the FBG + NaCl group rats. Our results illustrated that the inhibition of NLRP3 reversed myocardial fibrosis. Collectively, we demonstrated that NLRP3 inflammasome was activated after exposure to glucose fluctuations and may be responsible for promoting myocardial fibrosis.

NF-κB, a transcription factor, is involved in regulating oxidative stress, inflammation and apoptosis (19). Previous studies demonstrated that NF-κB activation was found in rats with glucose fluctuations (3, 5). In our study, the phosphorylation level of NF-κB/p65 was up-regulated in the FBG group. Moreover, nucleation of NF-κB/p65 was obviously observed in NRCFs cultured with normal/high glucose concentrations. Additionally, NF-κB activated by exposure to high glucose can promote inflammasome activation (20–22). In order to clarify the role of NF-κB in the glucose fluctuations-mediated inflammasome-involved regulation of myocardial fibrosis, the IKK-2 inhibitor TPCA-1 was applied in this study. TPCA-1 inhibits IKK-2 activity and decreases IκB phosphorylation, therefore IκB is not able to dissociate from NF-κB/p65 without phosphorylation, leading to preventing NF-κB/p65 nucleation. Furthermore, it has been reported that p65 could be bound to the NLRP3 promoter region and p65 silence resulted in decreased NLRP3 expression (14). Our data indicated that the inhibition of NF-κB/p65 nucleation reduced NLRP3 expression and NLRP3-ASC interaction, leading to reverse the effects of myocardial fibrosis induced by glucose fluctuations. Thus, we demonstrated that activation of NF-κB in response to glucose fluctuations could promote NLRP3 inflammasome assembling and aggravate myocardial fibrosis.

In summary, our research suggests that blood glucose fluctuations can aggravate NLRP3 inflammasome-induced myocardial fibrosis by activating NF-κB. Based on these results, glucose fluctuations may be a more important risk factor in the development of cardiac fibrosis and cardiac dysfunction than persistent hyperglycemia. Therefore, better monitoring of blood glucose in order to control glucose fluctuations and modulate inflammasome may provide a new method of clinical treatment to reverse diabetic myocardial fibrosis.

## Data Availability Statement

The original contributions presented in the study are included in the article/supplementary material, further inquiries can be directed to the corresponding authors.

## Ethics Statement

The animal study was reviewed and approved by Institutional Animal Care of the Affiliated Wuxi People’s Hospital of Nanjing Medical University.

## Author Contributions

R-XW, Z-YZ, and L-LQ were involved in the experiment design. Z-YZ, S-PD, S-SL, YL, M-MQ, and NW performed the experiments. Z-YZ, L-LQ, and L-FM analyzed the data. Z-YZ wrote the manuscript. YW, X-YL, and C-XW edited the manuscript. All authors read and approved the final manuscript.

## Conflict of Interest

The authors declare that the research was conducted in the absence of any commercial or financial relationships that could be construed as a potential conflict of interest.

## Publisher’s Note

All claims expressed in this article are solely those of the authors and do not necessarily represent those of their affiliated organizations, or those of the publisher, the editors and the reviewers. Any product that may be evaluated in this article, or claim that may be made by its manufacturer, is not guaranteed or endorsed by the publisher.
